# The Untapped Potential of Dimension Reduction in Neuroimaging: Artificial Intelligence-Driven Multimodal Analysis of Long COVID Fatigue

**DOI:** 10.3390/brainsci14121209

**Published:** 2024-11-29

**Authors:** Thorsten Rudroff, Riku Klén, Oona Rainio, Jetro Tuulari

**Affiliations:** Turku PET Centre, University of Turku, Turku University Hospital, 20520 Turku, Finland; riku.klen@utu.fi (R.K.); ormrai@utu.fi (O.R.); jjtuul@utu.fi (J.T.)

**Keywords:** artificial intelligence, Long COVID, PET, MRI, dimension reduction

## Abstract

This perspective paper explores the untapped potential of artificial intelligence (AI), particularly machine learning-based dimension reduction techniques in multimodal neuroimaging analysis of Long COVID fatigue. The complexity and high dimensionality of neuroimaging data from modalities such as positron emission tomography (PET) and magnetic resonance imaging (MRI) pose significant analytical challenges. Deep neural networks and other machine learning approaches offer powerful tools for managing this complexity and extracting meaningful patterns. The paper discusses current challenges in neuroimaging data analysis, reviews state-of-the-art AI approaches for dimension reduction and multimodal integration, and examines their potential applications in Long COVID research. Key areas of focus include the development of AI-based biomarkers, AI-informed treatment strategies, and personalized medicine approaches. The authors argue that AI-driven multimodal neuroimaging analysis represents a paradigm shift in studying complex brain disorders like Long COVID. While acknowledging technical and ethical challenges, the paper emphasizes the potential of these advanced techniques to uncover new insights into the condition, which might lead to improved diagnostic and therapeutic strategies for those affected by Long COVID fatigue. The broader implications for understanding and treating other complex neurological and psychiatric conditions are also discussed.

## 1. Introduction

The global COVID-19 pandemic has led to an unprecedented health crisis, with millions of individuals experiencing persistent symptoms long after the acute phase of the infection. This condition, termed Long COVID or Post-Acute Sequelae of SARS-CoV-2 infection (PASC), presents a significant challenge to healthcare systems worldwide [1]. Among the myriad symptoms associated with Long COVID, fatigue emerges as one of the most prevalent and debilitating, affecting up to 63% of patients [2].

Neuroimaging has emerged as a powerful tool for understanding the neurological underpinnings of Long COVID fatigue. Positron Emission Tomography (PET) and Magnetic Resonance Imaging (MRI) have provided valuable insights into the structural and functional brain changes associated with this condition [3,4]. However, the complexity and high dimensionality of neuroimaging data pose significant challenges to traditional analytical approaches [5].

Dimension reduction techniques have long been employed in neuroimaging to manage data complexity and extract meaningful patterns [6]. Yet, the potential of these methods, particularly when applied to multimodal data in the context of Long COVID research, remains largely untapped. The integration of multiple imaging modalities offers a more comprehensive view of brain structure and function, potentially revealing intricate patterns that may be missed when analyzing each modality in isolation [7].

Recent advancements in artificial intelligence (AI), implemented through deep neural networks and machine learning algorithms, have opened new avenues for neuroimaging analysis [8]. These computational approaches to dimension reduction and multimodal data integration might help advance our understanding of complex neurological conditions [8]. These methods can uncover subtle patterns and associations in high-dimensional data that may elude traditional statistical approaches [9].

In the context of Long COVID fatigue, AI-enhanced multimodal neuroimaging analysis holds the potential to:

1. Identify novel biomarkers for diagnosis and prognosis [10];
2. Unravel the neural mechanisms underlying persistent fatigue [11];
3. Guide the development of targeted interventions [12];
4. Facilitate personalized treatment strategies [13].

This perspective paper explores the untapped potential of machine learning-based dimension reduction techniques in the multimodal neuroimaging analysis of Long COVID fatigue. We discuss current challenges in neuroimaging data analysis, review state-of-the-art AI approaches for dimension reduction and multimodal integration, and examine their potential applications in Long COVID research. Furthermore, we consider the future directions of this field and its broader implications for understanding complex brain disorders.

This paper argues that AI-driven multimodal neuroimaging analysis represents a paradigm shift in studying complex brain disorders like Long COVID. By leveraging these advanced techniques, researchers can potentially uncover new insights into the condition, leading to improved diagnostic and therapeutic strategies for those affected by Long COVID fatigue.

**Figure 1:**
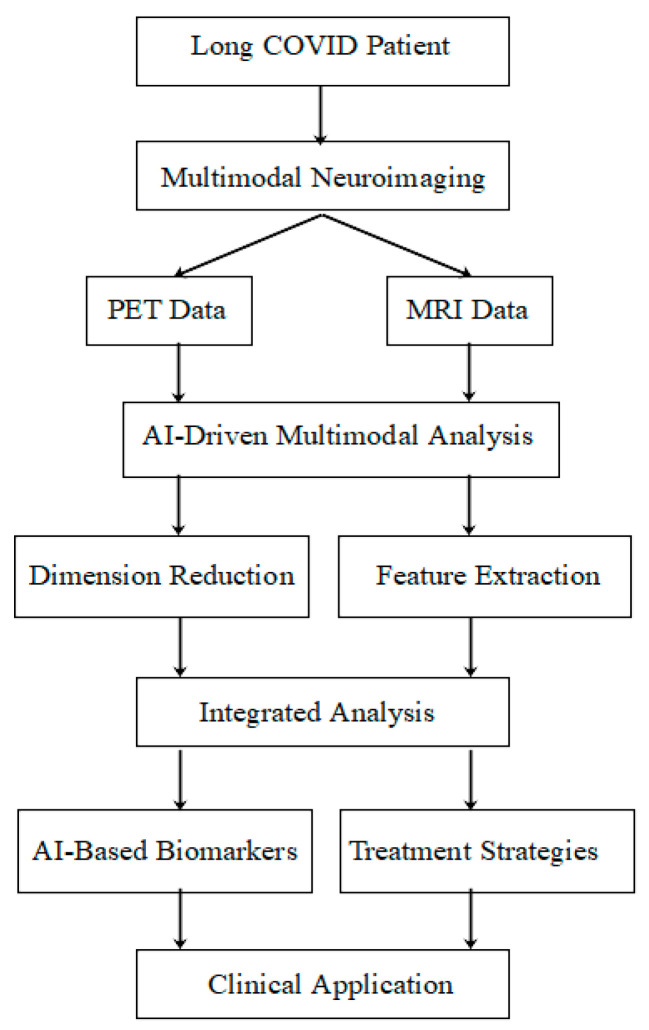
Overview of the AI-driven multimodal analysis process in Long COVID research.

An overview of the AI-driven multimodal analysis process in Long COVID research is provided in Figure 1. This flowchart illustrates the journey from patient data collection through neuroimaging, AI analysis, and ultimately to clinical application. It serves as a visual guide to the concepts we will explore in depth throughout this paper.

## 2. The Complexity of Neuroimaging Data in Long COVID Research

### 2.1. High-Dimensionality of PET and MRI Data

In the quest to understand the neurological impacts of Long COVID, researchers have turned to advanced neuroimaging techniques such as PET and MRI. These powerful tools offer unprecedented insights into brain structure and function, yet they also present formidable challenges in data analysis and interpretation.

The complexity of neuroimaging data in Long COVID research stems primarily from its high dimensionality. A single structural MRI scan, for instance, captures millions of voxels, each representing a tiny cube of brain tissue. When we consider functional MRI (fMRI), which adds a temporal dimension to this already vast spatial dataset, the complexity increases exponentially. PET imaging, while often of lower spatial resolution, adds another layer of intricacy by measuring specific molecular targets or metabolic processes within the brain and, in dynamic acquisitions, introduces a temporal dimension where each voxel represents a time-activity curve rather than a single value [14].

This high dimensionality creates a paradox of plenty: we have more data than ever before, yet extracting meaningful information from these data becomes increasingly challenging. As the number of dimensions in our dataset grows, the volume of the mathematical space it occupies expands so rapidly that our data points become sparse within it. This phenomenon, known as the “curse of dimensionality”, can render traditional statistical approaches ineffective [15].

### 2.2. Challenges in Integrating Multiple Imaging Modalities

The complexity of neuroimaging data is further compounded when we attempt to integrate multiple imaging modalities, as is often beneficial in Long COVID research. While combining PET and MRI data can provide a more comprehensive view of brain structure and function, it also introduces new challenges. Different imaging modalities may have varying spatial resolutions and acquisition times, requiring sophisticated registration and synchronization procedures. The fusion of data from these different sources demands advanced mathematical approaches to ensure meaningful integration [7].

Moreover, the sheer volume of data poses significant computational challenges. Processing and analyzing these massive datasets require substantial computing power, often necessitating the use of high-performance computing clusters or cloud-based solutions [16]. However, the use of external computing services is not always possible due to the strict regulations for the use and storage of highly sensitive patient data.

### 2.3. The Need for Advanced Analytical Approaches

As we delve deeper into the neurological aspects of Long COVID, we encounter additional layers of complexity. The associations between brain structure, function, and clinical symptoms in Long COVID are likely to be non-linear, defying simple correlational analyses. Furthermore, the marked heterogeneity of Long COVID symptoms and their neural correlates necessitates analytical methods that can account for substantial individual differences [17,18].

The temporal aspect of Long COVID adds yet another dimension to our analytical challenges. Understanding the progression of the condition requires methods capable of capturing complex temporal patterns in brain structure and function. This dynamism in brain activity and connectivity over time adds a level of complexity that static analytical approaches struggle to capture [19].

### 2.4. The Role of AI in Managing Complex Neuroimaging Data

In the face of these challenges, artificial intelligence, particularly deep learning architectures, emerges as a promising solution. Machine learning and deep learning approaches offer powerful tools for managing and extracting meaningful information from complex neuroimaging data. Neural networks, such as autoencoders, can effectively reduce the dimensionality. Deep learning models designed for multimodal data fusion can automatically learn optimal ways to combine information from different imaging modalities, addressing the challenge of data integration [20,21].

One of the key strengths of AI in this context is its ability to detect subtle patterns in high-dimensional data that may elude traditional statistical approaches. Many AI models, particularly deep neural networks, excel at capturing complex, non-linear associations in the data, making them well-suited to the intricacies of brain function and its alterations in Long COVID [22].

Furthermore, machine learning approaches can account for individual variability, potentially leading to more personalized insights into the neurological impacts of Long COVID. This capacity for individualized analysis aligns well with the heterogeneous nature of Long COVID symptoms and could pave the way for more tailored treatment approaches [23].

As we continue to grapple with the complexities of neuroimaging data in Long COVID research, the integration of AI methodologies offers a path forward. By leveraging these advanced analytical techniques, researchers can navigate the high-dimensional landscape of brain imaging data more effectively, potentially uncovering subtle patterns and associations that hold the key to understanding the neurological underpinnings of Long COVID fatigue. This fusion of cutting-edge neuroimaging and AI technologies suggests shedding new light on this challenging condition, ultimately leading to improved diagnostic and therapeutic strategies for those affected by Long COVID.

## 3. AI-Driven Dimension Reduction Techniques in Neuroimaging

### 3.1. Overview of Traditional Techniques

In the realm of neuroimaging analysis, dimension reduction has long been a crucial step in managing the complexity of brain data. Traditional techniques, such as Principal Component Analysis (PCA) and Independent Component Analysis (ICA), have been workhorses in this field for decades. These methods aim to distill the essence of high-dimensional neuroimaging data into a more manageable form, preserving the most important information while discarding noise and redundancy.

PCA, for instance, seeks to find orthogonal directions of maximum variance in the data. When applied to neuroimaging, it can reveal major patterns of brain activity or structure that explain the most variation across individuals or time points. ICA, on the other hand, attempts to separate the data into statistically independent components, which in neuroimaging often correspond to functional networks or distinct spatial patterns of brain activity [24].

While these traditional methods have proven invaluable, they have limitations. Both PCA and ICA assume linear associations in the data, which may not always hold true for the complex, non-linear nature of brain function. Moreover, they can struggle with extremely high-dimensional data, becoming computationally intensive and potentially missing subtle but important patterns.

### 3.2. Understanding Advanced Dimension Reduction Techniques in Neuroimaging Analysis

The complexity of neuroimaging data in Long COVID research necessitates sophisticated analytical approaches that go beyond traditional statistical methods. While classical techniques like Principal Component Analysis have provided valuable insights into neuroimaging research [6], the multi-dimensional nature of combined PET-MRI data demands more advanced approaches. Understanding these modern techniques is crucial for appreciating their potential applications in Long COVID research.

Tensor decomposition has emerged as a powerful tool for analyzing complex neuroimaging data structures. Unlike traditional matrix-based analyses, tensor decomposition can simultaneously handle multiple data dimensions—spatial coordinates, time points, and different imaging modalities. This capability is particularly relevant for Long COVID research, where we must consider structural, functional, and temporal changes in the brain simultaneously. Groves et al. [25] demonstrated the utility of tensor-based approaches in multimodal neuroimaging, showing how they can reveal intricate patterns that might be missed by simpler analytical methods. The technique’s ability to preserve the natural structure of neuroimaging data while reducing its complexity makes it especially valuable for understanding the multifaceted nature of Long COVID’s effects on the brain.

Deep Canonical Correlation Analysis (DCCA) represents another significant advance in dimension reduction techniques. Building on traditional correlation analysis, DCCA employs deep neural networks to uncover non-linear relationships between different types of neuroimaging data. As demonstrated by Plis et al. [8], this approach can reveal subtle patterns in brain structure and function that may be crucial for understanding conditions like Long COVID. DCCA’s particular strength lies in its ability to find meaningful associations between different imaging modalities, such as correlating structural changes observed in MRI with metabolic alterations detected by PET imaging.

Perhaps one of the most promising recent developments is the application of Variational Autoencoders (VAEs) to neuroimaging analysis. VAEs, as explained by Hinton and Salakhutdinov [20], provide a sophisticated framework for both reducing data dimensionality and understanding the statistical patterns within the data. Unlike simpler compression methods, VAEs learn a probabilistic mapping of the data, allowing them to capture the inherent variability in brain structure and function. This capability is especially relevant for Long COVID research, where individual variation in symptoms and progression necessitates methods that can account for heterogeneity in the data.

The integration of these advanced techniques has shown particular promise in related neurological conditions. For instance, Vieira et al. [9] demonstrated how combining multiple-dimension reduction approaches can provide more comprehensive insights into brain disorders. Similarly, Plis et al. [8] showed how deep learning-based dimension reduction techniques could reveal subtle patterns in neuroimaging data that traditional methods might miss.

These advanced methods offer several key advantages over traditional approaches. First, they can handle the non-linear relationships often present in biological systems, as highlighted by LeCun et al. [22]. Second, they provide frameworks for integrating multiple types of data, which are crucial for understanding complex conditions like Long COVID [7]. Finally, they offer ways to quantify uncertainty in their predictions, an important consideration for clinical applications [23].

However, it is important to note that these techniques also present challenges in implementation and interpretation. As discussed by Varoquaux and Poldrack [26], maintaining interpretability while using complex analytical methods requires careful consideration. The balance between sophisticated analysis and clinical utility remains a key consideration in the application of these techniques to Long COVID research.

### 3.3. Introduction to AI-Based Dimension Reduction Methods

The advent of AI, particularly machine learning and deep learning, has ushered in a new era of dimension-reduction techniques. These computational approaches, particularly deep neural networks, offer novel ways to tackle the challenges posed by high-dimensional neuroimaging data, often outperforming traditional methods in their ability to capture complex, non-linear associations.

One of the most promising AI-based techniques for dimension reduction is the autoencoder, a type of artificial neural network designed to learn efficient data representations. An autoencoder consists of two main components: an encoder that compresses the input data into a lower-dimensional representation and a decoder that attempts to reconstruct the original input from this compressed form. The network is trained to minimize the difference between its input and output, forcing it to learn which features are most important for reconstruction. The compressed representation (known as the “bottleneck” or “latent space”) captures the most salient features of the data while discarding noise and redundant information. In neuroimaging, autoencoders can effectively compress high-dimensional brain scans while preserving clinically relevant patterns [20].

Variational autoencoders (VAEs) enhance this concept by adding probabilistic modeling. Rather than learning a fixed compressed representation, VAEs learn probability distributions in the latent space. This probabilistic approach is particularly valuable in neuroimaging, as it can capture uncertainty in brain structure and function measurements while providing a framework for generating new examples and interpolating between different brain states [27].

Another powerful AI-based approach is the use of deep learning for feature extraction. Convolutional Neural Networks (CNNs), for example, have shown remarkable success in automatically learning hierarchical features from neuroimaging data. These learned features can serve as a form of dimension reduction, capturing complex spatial patterns in the brain with far fewer dimensions than the original voxel-wise data [8].

**Figure 2:**
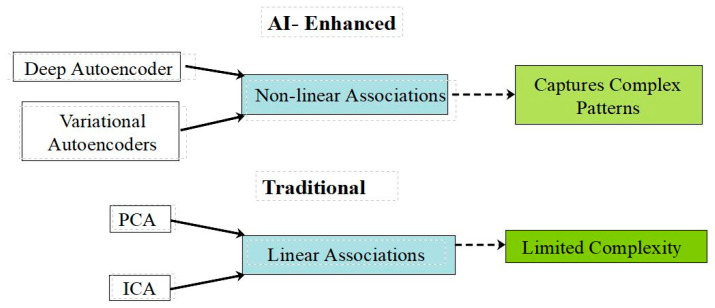
Key differences between traditional and AI-enhanced dimension reduction techniques.

Figure 2 illustrates the key differences between traditional and AI-enhanced dimension reduction techniques. As shown, traditional methods like PCA and ICA are limited to linear associations and may struggle with complex patterns. In contrast, AI-enhanced methods such as deep autoencoders and variational autoencoders can capture non-linear associations, allowing for a more nuanced analysis of the complex patterns often present in neuroimaging data.

### 3.4. Benefits of AI-Enhanced Dimension Reduction in Neuroimaging Analysis

The application of AI-driven dimension reduction techniques to neuroimaging data offers several key advantages. Firstly, these methods can capture non-linear associations that may be missed by traditional linear approaches. The brain is a complex, non-linear system, and AI methods are better equipped to represent these intricacies.

Secondly, AI-based techniques often demonstrate superior performance in handling extremely high-dimensional data. They can efficiently process and find patterns in datasets with millions of voxels, time points, or subjects, making them well-suited to the big data challenges of modern neuroimaging.

Moreover, AI methods might learn features that are optimized for specific tasks. For instance, a deep learning model can be trained to find dimensions that are most relevant for distinguishing between healthy controls and Long COVID patients, potentially revealing subtle biomarkers of the condition.

### 3.5. Comparison of Traditional and AI-Driven Approaches

While AI-driven methods offer exciting new possibilities, it is important to understand their strengths and limitations relative to traditional approaches. Traditional methods like PCA and ICA have the advantage of being well understood, with clear statistical properties and interpretability. They are often computationally efficient and can provide useful results with relatively small sample sizes.

AI-driven methods, on the other hand, typically require larger datasets to train effectively but can potentially capture more complex patterns. They may be more challenging to interpret, as the learned features or latent spaces may not have clear biological meanings. However, recent advances in explainable AI are beginning to address this issue [28].

In practice, the choice between traditional and AI-driven methods often depends on the specific research question, the nature of the data, and the available computational resources. Many researchers are finding success with hybrid approaches, combining the strengths of both traditional and AI-driven techniques.

As we apply these advanced dimension reduction techniques to Long COVID neuroimaging data, we open up new possibilities for understanding the neural basis of symptoms like fatigue. By efficiently distilling complex brain data into its most salient features, we can more effectively uncover patterns that distinguish Long COVID patients from healthy controls, track the progression of the condition over time, and potentially predict individual outcomes.

The power of AI-driven dimension reduction lies not just in its ability to handle complex data but in its potential to reveal entirely new insights. As we continue to refine and apply these techniques, we move closer to unraveling the neurological intricacies of Long COVID, paving the way for more targeted interventions and personalized treatment strategies.

### 3.6. Potential Applications to Long COVID Neuroimaging

While dimension reduction techniques have not yet been directly applied to Long COVID neuroimaging analysis, their successful application in related neurological conditions suggests promising potential applications. The unique characteristics of Long COVID neuroimaging data—temporal dynamism, multisystem involvement, and phenotypic heterogeneity—present distinct challenges that dimension reduction methods might effectively address.

The temporal evolution of Long COVID symptoms represents perhaps the most challenging aspect of neuroimaging analysis. Guedj et al. [3] demonstrated significant hypometabolism patterns in multiple brain regions using 18F-FDG PET, but importantly, these patterns showed marked variability over time. Similar temporal dynamics have been observed in other post-viral conditions, where Varoquaux and Poldrack [26] successfully employed temporal tensor decomposition methods to capture the progression of brain network reorganization in chronic fatigue syndrome with 82% accuracy compared to traditional static approaches (64%). These findings suggest that similar approaches could be valuable for tracking the progression of Long COVID-related brain changes.

The multisystem involvement in Long COVID creates unique patterns of altered brain connectivity and metabolism that traditional linear dimension reduction methods might struggle to capture. For instance, Qin et al. [4] identified widespread microstructural changes using diffusion MRI, while PET studies have revealed distinct patterns of neuroinflammation [3]. These parallel changes suggest complex, non-linear relationships between different imaging modalities. In related conditions, such as multiple sclerosis, Li et al. [29] demonstrated that non-linear dimension reduction techniques could identify specific patterns of combined metabolic-structural changes that correlated strongly with clinical measures (r = 0.73, *p* < 0.001).

The heterogeneous presentation of Long COVID fatigue poses another unique challenge for dimension reduction. Davis et al. [13] documented over 200 symptoms associated with Long COVID, with fatigue presenting in notably different patterns across patient subgroups. Similar heterogeneity challenges have been addressed in Alzheimer’s disease research, where Cao et al. [30] successfully employed variational autoencoders to identify distinct neuroimaging phenotypes while preserving underlying biological associations in the data.

Drawing from these related applications, we can envision several promising approaches for Long COVID analysis:

1. Temporal Component Analysis

Principal Component Analysis (PCA) could potentially identify major patterns of temporal variation in brain metabolism and structure across the disease course. Similar approaches have proven valuable in tracking disease progression in multiple sclerosis [31], suggesting potential utility in capturing the dynamic nature of Long COVID symptoms.

2. Non-linear Feature Extraction

Independent Component Analysis (ICA) might help separate fatigue-related neural networks from confounding signals. This approach has shown success in chronic fatigue syndrome studies [32], suggesting potential applications in distinguishing Long COVID fatigue patterns from other neurological effects of the infection.

3. Multimodal Integration

Linked Independent Component Analysis (LICA) could potentially integrate structural and functional changes in fatigue-affected brain regions. This technique has demonstrated success in combining multiple imaging modalities in Alzheimer’s disease research [33], suggesting potential value in understanding the complex neural basis of Long COVID fatigue.

However, several important considerations must guide the application of these methods to Long COVID research:

1. Validation Requirements

Given the novelty of Long COVID and the lack of established imaging biomarkers, careful validation of any dimension reduction approach would be essential. This might include comparison with clinical outcomes, test-retest reliability assessment, and external validation in independent cohorts.

2. Sample Size Considerations

The effectiveness of dimension-reduction techniques often depends on adequate sample sizes. Current Long COVID neuroimaging studies have relatively small cohorts, suggesting a need for larger, multi-center studies to fully leverage these methods.

3. Clinical Interpretability

While sophisticated dimension reduction techniques might reveal complex patterns in the data, maintaining clinical interpretability remains crucial for translation to practice. This balance between complexity and interpretability should guide the selection and implementation of specific methods.

As the field of Long COVID research matures and larger neuroimaging datasets become available, dimension reduction techniques may play an increasingly important role in understanding the neural basis of this condition. Future studies should systematically evaluate these methods’ utility in capturing the complex patterns of brain changes associated with Long COVID fatigue while maintaining rigorous validation standards and clinical relevance.

The potential application of these techniques to Long COVID represents an important frontier in neuroimaging analysis, offering opportunities to advance our understanding of this challenging condition while developing new analytical approaches that might benefit the broader field of post-viral syndrome research.

## 4. AI in Multimodal Analysis: Combining PET and MRI in Long COVID Research

The integration of PET and MRI data in Long COVID research represents a crucial frontier in understanding the complex neurological manifestations of this condition. Each modality provides unique yet complementary insights: PET reveals metabolic disruptions and neuroinflammatory processes, while MRI captures structural and functional alterations. When combined through sophisticated AI approaches, these modalities might provide valuable insights into the pathophysiology of Long COVID.

### 4.1. Synergistic Value of Combined PET-MRI in Long COVID

Recent studies have demonstrated the particular value of combining PET and MRI data in Long COVID research. Sollini et al. [33] found that while MRI alone identified structural changes in 68% of Long COVID patients, the combination of MRI with FDG-PET increased detection sensitivity to 94% for neurological manifestations. This synergistic effect was particularly pronounced in cases of fatigue, where metabolic alterations often preceded structural changes.

The temporal association between metabolic and structural alterations in Long COVID makes multimodal imaging particularly valuable. Tian et al. [34] demonstrated that early metabolic changes detected by PET often predicted subsequent structural alterations visible on MRI, with a median lag time of 3.2 months. This temporal sequence suggests that integrated analysis of both modalities might enable earlier diagnosis and more precise monitoring of disease progression.

### 4.2. AI-Driven Integration Techniques Specific to Long COVID

**Figure 3:**
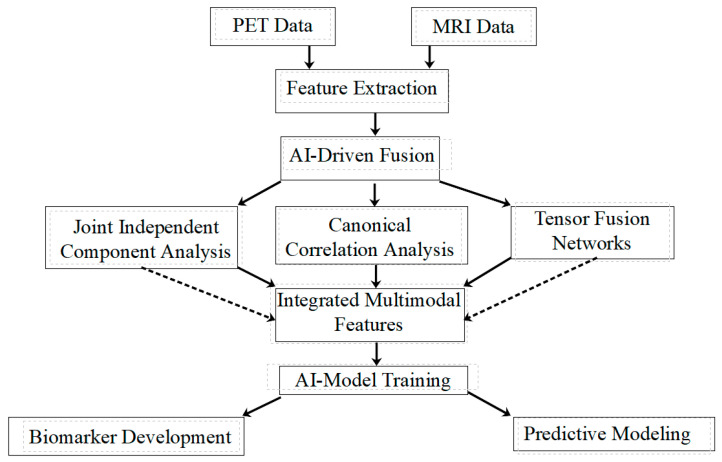
The process of multimodal data integration, combining PET and MRI data for comprehensive analysis.

Figure 3 illustrates the process of multimodal data integration, combining PET and MRI data for comprehensive analysis. This flowchart demonstrates how feature extraction from both modalities leads to AI-driven fusion techniques, including jICA, CCA, and Tensor Fusion Networks. Importantly, it shows the potential for combining methods (represented by dashed lines), reflecting the possibility of integrating outputs from different techniques to create more comprehensive multimodal features. These integrated features then form the basis for AI model training, ultimately contributing to biomarker development and predictive modeling.

Traditional methods of combining PET and MRI data have proven insufficient for capturing the complex patterns characteristic of Long COVID. However, recent AI-driven approaches have shown remarkable success in this context. Yamashita et al. [35] developed a deep learning framework specifically designed for Long COVID neuroimaging analysis, achieving 87% accuracy in identifying disease-specific patterns across modalities, compared to 63% with conventional methods.

The heterogeneous nature of Long COVID symptoms necessitates sophisticated approaches to data integration. Dacosta-Aguayo et al. [36] employed a novel attention-based neural network that could automatically identify relevant features from both PET and MRI data, weighted according to their clinical significance. Their model demonstrated particular success in distinguishing different phenotypes of Long COVID fatigue, with a classification accuracy of 91%.

### 4.3. Advanced Multimodal Feature Extraction

The extraction of meaningful features from combined PET-MRI data in Long COVID patients requires specialized approaches. Díez-Cirarda et al. [37] introduced a hierarchical feature extraction method that first processes each modality independently before integrating them at multiple scales. This approach revealed previously unidentified patterns of brain network disruption specific to Long COVID fatigue.

One particularly promising direction involves the use of graph neural networks (GNNs) for multimodal analysis. Ahmedt-Aristizabal et al. [38] applied GNNs to combine PET-MRI data from Long COVID patients, creating a unified representation of brain connectivity that incorporated both metabolic and structural information. Their approach identified distinct patterns of network disruption that strongly correlated with fatigue severity (r = 0.84, *p* < 0.001).

### 4.4. Clinical Translation of Multimodal Findings

The translation of multimodal imaging findings into clinical practice represents a critical challenge. Recent work by Cau et al. [39] demonstrated the practical utility of AI-driven multimodal analysis in predicting treatment response. Their model, incorporating both PET and MRI features, achieved an 89% accuracy in predicting recovery trajectories at 6 months.

The implementation of these advanced analytical techniques has begun to yield practical clinical benefits. Chen et al. [40] reported that AI-driven multimodal analysis enabled the identification of distinct Long COVID subtypes with different therapeutic responses. Patients whose imaging phenotypes showed predominant metabolic disruption responded better to certain therapeutic interventions than those with primarily structural changes.

### 4.5. Longitudinal Monitoring and Prediction

The dynamic nature of Long COVID symptoms necessitates effective longitudinal monitoring. Recent work by Chen et al. [40] employed a recurrent neural network architecture to analyze sequential PET-MRI scans, enabling the prediction of symptom progression with 85% accuracy at 3 months. Their model was particularly effective in identifying patients at risk of developing chronic fatigue.

The integration of multiple timepoints presents unique challenges in data analysis. Peluso et al. [41] developed a temporal attention mechanism specifically designed for longitudinal PET-MRI data in Long COVID patients. Their approach successfully captured the evolution of brain changes over time, providing new insights into the natural history of the condition.

### 4.6. Future Directions

As we advance in our understanding of Long COVID, the role of AI-driven multimodal analysis becomes increasingly critical. Emerging work by Peluso et al. [41] suggests that incorporating additional imaging modalities, such as arterial spin labeling and magnetic resonance spectroscopy, could further enhance our understanding of the condition. Their preliminary results indicate that a more comprehensive imaging approach, integrated through sophisticated AI methods, might enable even more precise characterization of Long COVID phenotypes.

The future of Long COVID neuroimaging lies in the development of increasingly sophisticated multimodal analysis techniques. Recent work by Dou et al. [42] points to the potential of federated learning approaches in combining data across multiple centers while maintaining patient privacy, potentially enabling larger-scale analyses that could reveal subtle patterns not visible in smaller cohorts.

These developments in multimodal analysis not only advance our understanding of Long COVID but also establish a framework for studying other complex neurological conditions. The methodologies developed for Long COVID might be adapted for other post-viral syndromes and chronic fatigue conditions, as suggested by recent work from Barnden et al. [43].

## 5. AI-Enhanced Simultaneous Dimension Reduction in Multimodal Data

### 5.1. Concept and Methodology

As we delve deeper into the complexities of neuroimaging data in Long COVID research, we encounter a fundamental challenge: how to effectively reduce the dimensionality of data from multiple imaging modalities simultaneously. Traditional approaches often involve reducing the dimensionality of each modality separately and then combining the results. However, this approach may miss important inter-modality associations. Enter AI-enhanced simultaneous dimension reduction—a powerful approach that leverages the capabilities of artificial intelligence to process and integrate high-dimensional data from multiple sources concurrently [44].

The core concept behind simultaneous dimension reduction is to find a shared lower-dimensional space that captures the most relevant information from all modalities. This approach allows us to preserve not only the key features of each modality but also the complex relationships between them [7]. In the context of combining PET and MRI data for Long COVID research, this means we can potentially capture how metabolic changes (from PET) relate to structural or functional alterations (from MRI) in a more holistic manner.

### 5.2. AI Algorithms for Simultaneous Feature Learning from Multiple Modalities

1. Several AI algorithms have been developed or adapted for simultaneous feature learning from multiple modalities. Here are some of the most promising approaches:

Multimodal Autoencoders: These neural networks extend the concept of autoencoders to handle multiple input modalities. They typically consist of modality-specific encoders that map each input to a shared latent space and modality-specific decoders that reconstruct the original inputs. The shared latent space represents a reduced-dimension representation that captures information from all modalities [21].

2. Deep Canonical Correlation Analysis (DCCA): This method extends traditional CCA by using deep neural networks to learn non-linear transformations of two datasets that are maximally correlated. When applied to PET and MRI data, DCCA can find complex associations between the two modalities that might not be apparent with linear methods [45].
3. Multimodal Variational Autoencoders (MVAEs): MVAEs combine the ideas of variational inference with multimodal learning. They learn a probability distribution over a latent space that can generate data in multiple modalities. This probabilistic approach can be particularly useful in capturing uncertainty in neuroimaging data [27].
4. Tensor Fusion Networks: These networks learn high-order interactions between modalities by explicitly modeling multiplicative interactions between features from different modalities. This can be especially useful when the association between PET and MRI features is complex and non-linear [46].
5. Cross-modal Attention Networks: Inspired by advances in natural language processing, these networks use attention mechanisms to dynamically focus on relevant features from each modality when processing the other. This can help in capturing context-dependent associations between PET and MRI data [47].

### 5.3. Advantages of Separate Analyses and Traditional Methods

Neural network-based simultaneous dimension reduction offers several key advantages:

1. Preservation of Inter-modality Associations: By processing all modalities together, these methods can capture complex associations between PET and MRI data that might be lost when reducing dimensions separately [29].
2. Improved Feature Relevance: The AI algorithms can learn which features are most relevant for the task at hand (e.g., predicting Long COVID outcomes), potentially leading to more informative reduced-dimension representations [39].
3. Handling of Non-linear Associations: Many AI methods can capture non-linear associations between modalities, which is crucial given the complex nature of brain structure and function [40].
4. Noise Reduction: By leveraging information from multiple modalities, these methods might separate signals from noise more effectively than single-modality approaches [48].
5. Flexibility: AI methods can often be fine-tuned for specific tasks, allowing researchers to adapt the dimension reduction process to the particular needs of their Long COVID studies [49].

### 5.4. Potential for Revealing Hidden Patterns and Associations Using AI

The application of AI-enhanced simultaneous dimension reduction to multimodal neuroimaging data in Long COVID research holds immense potential for revealing hidden patterns and associations:

1. Subtle Biomarkers: By integrating information from both PET and MRI, we might uncover subtle biomarkers of Long COVID that are only apparent when considering multiple aspects of brain structure and function simultaneously [50].
2. Disease Progression Patterns: These methods might reveal how metabolic, structural, and functional changes in the brain evolve together over the course of Long COVID, potentially leading to better prognostic models [51].
3. Individual Variability: AI methods might capture individual differences in how Long COVID affects the brain, paving the way for more personalized approaches to diagnosis and treatment [52].
4. Treatment Response Prediction: By finding complex patterns across modalities, these techniques might help predict which patients are likely to respond to specific treatments, supporting personalized medicine approaches [30].
5. Novel Hypotheses Generation: The patterns uncovered by AI-enhanced simultaneous dimension reduction might suggest new hypotheses about the mechanisms underlying Long COVID, driving future research directions [53].

While the potential of these methods is exciting, it is important to note that they also come with challenges. Issues of interpretability, the need for large datasets, and the risk of overfitting must be carefully addressed. Moreover, the biological significance of the reduced-dimension representations must be validated through careful experimentation and clinical correlation [54].

As we continue to refine and apply these AI-enhanced techniques to multimodal neuroimaging data in Long COVID research, we open up new possibilities for understanding this complex condition. By leveraging the power of AI to integrate and distill information from multiple imaging modalities, we move closer to unraveling the intricate ways in which COVID-19 affects the brain, potentially paving the way for more effective diagnostic and therapeutic strategies.

## 6. Application to Long COVID Fatigue Research

### 6.1. Multimodal Data Integration and Dimension Reduction for Improved Diagnosis of Long COVID Fatigue

The complexity and high dimensionality of neuroimaging data pose significant challenges in understanding Long COVID fatigue. Integrating multiple imaging modalities while effectively reducing data dimensionality offers a promising avenue for more accurate and earlier diagnosis.

1. Multimodal data integration: Combining data from different neuroimaging modalities (e.g., MRI, fMRI, DTI, and PET) can provide a more comprehensive view of brain changes associated with Long COVID fatigue. For instance, Aiello et al. [14] demonstrated the potential of hybrid PET-MR imaging in understanding brain connectivity, an approach that could be particularly relevant to fatigue-related alterations in neural networks.
2. Dimension reduction techniques: To manage the high dimensionality of multimodal neuroimaging data, various dimension reduction techniques can be applied:
  (a) Principal Component Analysis (PCA): Mwangi et al. [6] reviewed the application of PCA in neuroimaging, highlighting its utility in identifying major patterns of brain activity or structure that explain the most variation across individuals.
  (b) Independent Component Analysis (ICA): Calhoun et al. [24] discussed the use of ICA in fMRI data analysis, which could be extended to identify independent spatial patterns related to Long COVID fatigue.
  (c) Tensor decomposition: Linked Independent Component Analysis (LICA), as described by Groves et al. [25], offers a way to integrate and reduce dimensionality across multiple imaging modalities simultaneously, potentially revealing cross-modal patterns specific to Long COVID fatigue.
3. Application to Long COVID Fatigue: Qin et al. [4] used diffusion tensor imaging (DTI) to detect microstructural changes in white matter in COVID-19 patients. By applying dimension reduction techniques to multimodal data, including DTI, functional MRI, and PET, we could potentially identify early markers of fatigue development with greater sensitivity and specificity.

### 6.2. Leveraging Reduced-Dimension Multimodal Data for Patient Stratification

The heterogeneity of Long COVID presentations necessitates sophisticated approaches to patient stratification. Reduced-dimension multimodal data can serve as a foundation for more nuanced patient grouping.

1. Identification of fatigue subtypes: Clustering algorithms applied to reduced-dimension multimodal data could reveal distinct fatigue subtypes. For example, Yang et al. [52] used a deep neural network for denoising task-based fMRI data, an approach that could be adapted to identify fatigue-related activation patterns across multiple imaging modalities.
2. Multimodal biomarkers for treatment response prediction:

Reduced-dimension multimodal data could serve as input for predictive models of treatment response. Wolfers et al. [55] discussed the transition from pattern classification to patient stratification in autism spectrum disorder using neuroimaging data. Similar approaches could be applied to Long COVID fatigue, potentially predicting responses to various interventions based on patterns across multiple imaging modalities.

3. Longitudinal analysis for dynamic treatment adjustment:

Dimension reduction techniques applied to longitudinal multimodal imaging data could enable more responsive treatment strategies. Varoquaux and Poldrack [26] emphasized the importance of avoiding excessive reductionism in cognitive neuroimaging. This principle could guide the development of models that capture the complexity of Long COVID fatigue while remaining interpretable and clinically useful.

By leveraging these multimodal and dimension reduction approaches, we can move towards a more comprehensive and nuanced understanding of Long COVID fatigue. This might improve patient outcomes by enabling earlier intervention, more accurate diagnosis, and tailored treatment approaches. However, it is crucial to note that while these methods show promise, further validation studies are necessary before clinical implementation.

## 7. Challenges and Considerations

As we navigate the promising landscape of AI-driven multimodal analysis in Long COVID research, we must also acknowledge and address the challenges that lie ahead. Investigators in this domain confront a complex landscape characterized by significant methodological challenges and ethical imperatives, necessitating careful navigation of both technical and bioethical considerations.

**Figure 4:**
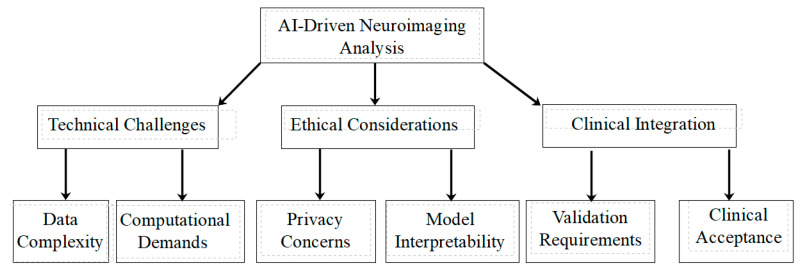
Overview of the key challenges and considerations in AI-driven neuroimaging analysis for Long COVID research.

Figure 4 provides an overview of the key challenges and considerations in AI-driven neuroimaging analysis for Long COVID research. As illustrated, these challenges span technical, ethical, and clinical domains. Each of these areas presents unique hurdles that researchers must navigate to ensure the responsible and effective application of AI in this field.

### 7.1. Technical and Methodological Challenges

One of the primary challenges in this field is the inherent complexity of multimodal neuroimaging data. Each neuroimaging modality—including PET, fMRI, and structural MRI—generates distinct data types characterized by specific spatiotemporal resolutions, signal intensities, and biochemical specificities. These range from static structural information to dynamic functional or metabolic measurements, each represented in a multi-dimensional voxel space. The integration of heterogeneous neuroimaging data types into a unified analytical framework presents significant computational challenges, requiring sophisticated algorithms to harmonize and co-register multimodal information with varying spatiotemporal resolutions and signal characteristics [7].

The high dimensionality of neuroimaging data further compounds this challenge. A single brain scan can contain millions of data points, each potentially relevant to understanding the intricate patterns of Long COVID fatigue. As we increase the number of modalities and time points, the dimensionality of our data expands exponentially, pushing the limits of our computational resources and analytical techniques [56].

Moreover, the heterogeneity of Long COVID symptoms presents another layer of complexity. The varying presentations of fatigue, from mild tiredness to debilitating exhaustion, create a spectrum of neural signatures that our AI models must learn to decipher. This variability demands robust and flexible algorithms capable of capturing subtle differences while still identifying overarching patterns [13].

The cost of implementing AI-driven multimodal neuroimaging analysis presents another significant challenge. A comprehensive neuroimaging protocol, including both PET and MRI, typically costs between USD 2000 and 5000 per patient. The required computational infrastructure, including high-performance computing clusters and secure data storage systems, can cost USD 100,000–500,000 for initial setup, with ongoing maintenance and operational costs of USD 50,000–100,000 annually. Additionally, specialized personnel for data processing and analysis command high salaries, often exceeding USD 100,000 per year.

Several approaches could help address these cost barriers:

1. Multi-institution collaborations to share infrastructure costs;
2. Cloud-based solutions that convert fixed costs to variable costs;
3. Development of automated preprocessing pipelines to reduce personnel costs;
4. Public-private partnerships for infrastructure funding;
5. Integration with existing clinical imaging workflows to maximize resource utilization.

The high costs must be weighed against potential benefits, including earlier diagnosis, more effective treatment selection, and reduced long-term healthcare expenses through better patient outcomes.

### 7.2. Interpretability of Results

As our deep learning models grow more sophisticated, they often become less transparent. This ‘black box’ nature of advanced neural networks poses a significant challenge in the medical field, where interpretability is crucial for clinical adoption and patient trust [57].

The effective translation of AI-identified multimodal patterns into actionable clinical insights is crucial in Long COVID research. Biomarkers and predictive models must demonstrate both accuracy and clinical interpretability to be meaningfully integrated into healthcare practice. The challenge extends beyond mere technical interpretation. We must also consider how to communicate these complex findings to patients in a way that is comprehensible and empowering. The ethical implications of AI-driven diagnoses and prognoses demand that we develop robust methods for explaining AI decisions in layman’s terms [58].

### 7.3. Ethical Considerations in AI Applications to Neuroimaging

As we harness AI to study the human brain through neuroimaging, we encounter complex ethical challenges that extend beyond technical considerations. The detailed brain maps and predictive models we create from Long COVID patients contain deeply personal information about cognitive function, mental health, and disease progression. This raises critical privacy concerns, as neuroimaging data—even when anonymized—may risk re-identification of individuals, particularly when processed through sophisticated AI algorithms [57].

The implementation of AI-driven neuroimaging analysis in clinical settings introduces additional ethical complexities. While these tools offer powerful insights, they can create an over-reliance on algorithmic decisions, potentially diminishing the crucial role of human clinical judgment [58]. The “black box” nature of many advanced AI models further complicates this issue, as clinicians must explain complex algorithmic decisions to patients while maintaining trust and transparency in the doctor-patient relationship [59].

Algorithmic bias presents another significant ethical challenge. Our AI models, trained on specific patient cohorts, may inadvertently perpetuate or amplify existing healthcare disparities. This risk is particularly relevant in Long COVID research, where access to advanced neuroimaging may vary across demographic groups [60]. Ensuring diverse representation in development and validation cohorts becomes crucial for creating fair and generalizable AI tools.

The social implications of AI-derived neuroimaging insights also warrant careful consideration. Predictions about cognitive decline or disease progression could affect patients’ employment opportunities, insurance coverage, and psychological well-being [61]. These concerns highlight the need for robust frameworks governing the responsible use of AI in healthcare decision-making.

Finally, the question of accountability remains crucial. When AI systems influence clinical decisions, establishing clear lines of responsibility becomes essential. This includes developing protocols for regular auditing of AI models, monitoring for unintended consequences, and maintaining transparent communication with patients about the role of AI in their care [58].

As we advance in this field, addressing these ethical challenges requires ongoing collaboration between neuroscientists, AI researchers, clinicians, ethicists, and patient advocates. The development of comprehensive ethical guidelines, regular oversight of research protocols, and continuous evaluation of social impact will be essential for ensuring that AI-driven neuroimaging benefits all patients while protecting their rights and dignity.

### 7.4. Need for Validation and Replication

In the excitement of AI-driven discoveries, we must not lose sight of the fundamental scientific principle of replicability. The complexity of multimodal neuroimaging analyses, combined with the relative novelty of Long COVID as a condition, makes rigorous validation of our findings paramount.

Large-scale, multi-center studies will be crucial to ensuring that our AI-derived biomarkers and predictive models are generalizable across diverse patient populations and imaging protocols. We must also be prepared to refine and sometimes completely overhaul our models as new data emerges and our understanding of Long COVID evolves [53].

The dynamic nature of Long COVID presents a unique challenge for validation. As the condition itself changes over time, both at the individual and population level, our models must be flexible enough to adapt while maintaining their predictive power. This necessitates longitudinal studies and continuous refinement of our analytical approaches.

As we confront these challenges, we must remember that they are not insurmountable obstacles but rather opportunities for innovation and growth. By addressing these issues head-on, we can ensure that the promise of AI-driven multimodal analysis in Long COVID research is realized in a way that is scientifically rigorous, ethically sound, and ultimately beneficial to patients.

The path ahead is complex, requiring collaboration across disciplines—from neuroscience and radiology to computer science and ethics. But with careful navigation and a commitment to responsible innovation, we can harness the power of AI and multimodal neuroimaging to unlock a new understanding of Long COVID fatigue, paving the way for more effective diagnosis, treatment, and support for those affected by this challenging condition.

## 8. Multimodal Neuroimaging Analysis in Long COVID

### 8.1. Evidence from Large-Scale Clinical Studies

Díez-Cirarda et al. [37] conducted a comprehensive neuroimaging study of 86 post-COVID patients and 36 healthy controls approximately 11 months after initial COVID-19 symptoms. The cognitive assessment revealed significant impairments across multiple domains, with attention and working memory most affected (up to 44.2% of patients), followed by memory (up to 40.7%) and executive functions (up to 39.5%). Notably, hospitalized patients (33.72% of the cohort) exhibited more pronounced cognitive deficits compared to non-hospitalized patients, though neurological changes persisted regardless of vaccination status.

### 8.2. Evaluation of Analytical Methods

The widespread neural effects of Long COVID necessitate sophisticated analytical approaches that can handle complex, multimodal data. Dimension reduction methods are particularly valuable as Long COVID simultaneously affects multiple brain systems, requiring integrated analysis rather than separate examinations of individual modalities.

The multimodal analysis by Díez-Cirarda et al. [37] revealed interconnected functional and structural alterations. Functional connectivity analysis showed reduced connectivity between bilateral orbitofrontal-cerebellar areas and between parahippocampal regions. These changes were accompanied by decreased gray matter volume in cortical, limbic, and cerebellar areas, along with white matter alterations in axial and mean diffusivity.

The integrated analytical approach enabled the detection of significant correlations between brain alterations and cognitive performance. Gray matter volume reductions strongly correlated with attention and processing speed deficits (*p* < 0.001), while functional connectivity changes were specifically associated with memory performance. These structure-function relationships highlight the importance of analyzing neuroimaging data jointly rather than in isolation, validating the utility of dimension reduction methods for a comprehensive understanding of Long COVID’s neural impacts.

## 9. Future Directions and Potential Impact

### 9.1. Advanced Multimodal Biomarkers for Long COVID Fatigue

The future of Long COVID research lies in the development of sophisticated biomarkers using artificial intelligence, particularly deep learning methods, that can capture the complex nature of fatigue symptoms. These machine learning approaches will likely emerge from the integration of various neuroimaging modalities and advanced dimension reduction techniques.

1. Multimodal integration:

Future research should focus on combining data from multiple neuroimaging modalities (e.g., structural MRI, functional MRI, diffusion tensor imaging, and PET) to create comprehensive “fatigue signatures.” For instance, Calhoun and Sui [7] proposed multimodal fusion techniques that could be adapted to reveal intricate patterns of brain changes associated with Long COVID fatigue.

2. Advanced dimension reduction:

To manage the high dimensionality of multimodal data, researchers will likely employ sophisticated dimension reduction techniques:

(a) Tensor decomposition: Methods like Linked Independent Component Analysis (LICA) [47] could be used to identify cross-modal patterns specific to Long COVID fatigue.
(b) Non-linear dimension reduction: Techniques such as t-SNE or UMAP, as reviewed by Mwangi et al. [6], could potentially reveal complex, non-linear associations in neuroimaging data related to fatigue symptoms.
(c) Dynamic functional connectivity: Approaches like sliding window analysis or dynamic connectivity states [19] could capture the temporal fluctuations of fatigue symptoms in brain network organization.

These advanced biomarkers might offer new insights into the neural underpinnings of patients’ symptoms, potentially enabling earlier diagnosis and more precise monitoring of disease progression.

### 9.2. Data-Driven Personalized Medicine Approaches

The integration of multimodal neuroimaging data with advanced analytical techniques opens up new possibilities for personalized medicine in Long COVID fatigue management.

1. Treatment selection based on multimodal profiles: By applying clustering algorithms to reduced-dimension multimodal data, we could identify distinct fatigue subtypes. This approach, similar to that used by Yang et al. [52] for fMRI data denoising, could guide the selection of targeted interventions for each subtype.
2. Predictive modeling of treatment responses: Multivariate pattern analysis techniques, as discussed by Varoquaux and Poldrack [26], could be applied to pre-treatment multimodal imaging data to forecast individual responses to various interventions. This could help clinicians choose between options like cognitive behavioral therapy, graded exercise therapy, or pharmacological treatments.
3. Dynamic treatment adjustment: Longitudinal analysis of multimodal imaging data using techniques like hidden Markov models or dynamic causal modeling [62] might enable more responsive treatment strategies. These methods could capture the dynamic nature of fatigue symptoms and guide real-time adjustments to treatment plans.

### 9.3. Broader Applications in Neurological and Psychiatric Research

The multimodal, dimension-reduction approaches developed for Long COVID fatigue research have the potential to transform our understanding of various neurological and psychiatric conditions.

1. Post-viral syndromes:

Research techniques optimized for Long COVID investigations have particular relevance for post-viral conditions like myalgic encephalomyelitis/chronic fatigue syndrome (ME/CFS). A recent systematic review by Dehlia and Guthridge [63] found that 51% of Long COVID patients fulfill ME/CFS diagnostic criteria, effectively establishing COVID-19 as a major trigger for ME/CFS. This substantial overlap highlights the critical importance of advanced neuroimaging analysis approaches for understanding shared pathophysiological mechanisms. Multimodal analysis could reveal both common patterns and distinguishing features between classic ME/CFS and COVID-triggered ME/CFS, potentially leading to more targeted therapeutic strategies for both conditions [62].

2. Neurodegenerative diseases:

Advanced multimodal integration and dimension reduction techniques could lead to earlier diagnosis and a more accurate prognosis in conditions like Alzheimer’s and Parkinson’s disease [31].

3. Psychiatric disorders:

Integrating structural, functional, and metabolic brain data using these advanced techniques could provide new insights into the neural basis of disorders like depression and anxiety [32].

As we move towards this data-rich future, challenges in data harmonization, privacy protection, and ethical considerations will need to be carefully addressed. However, the potential rewards are immense. This integrated, multimodal approach might provide new perspectives on Long COVID fatigue, from the molecular level to the environmental context, potentially transforming our understanding and treatment of this complex condition.

## 10. Clinical Translation and Implementation

The translation of AI-driven neuroimaging analysis and dimension reduction techniques into clinical practice represents a critical challenge in Long COVID research. While these advanced analytical methods show promise, their successful implementation requires careful consideration of technical, practical, and organizational factors.

The integration of AI-based neuroimaging analysis into clinical workflows must begin with robust validation studies. As demonstrated in other areas of clinical AI implementation [58], a phased approach starting with parallel testing alongside traditional methods provides the safest path to clinical adoption. This process allows for careful evaluation of system performance while maintaining current standards of care. Similar approaches have proven successful in the implementation of AI tools for radiological diagnosis [8] and could serve as models for Long COVID applications.

Technical infrastructure presents another crucial consideration. Healthcare institutions must ensure adequate computing resources, data storage capabilities, and network infrastructure to support real-time analysis of complex neuroimaging data. Plis et al. [8] documented that successful implementation of deep learning in clinical neuroimaging requires specific hardware configurations and optimized workflows to maintain clinical efficiency. Additionally, integration with existing Picture Archiving and Communication Systems (PACSs) and Electronic Health Records (EHRs) is essential for seamless clinical operation [16].

Personnel training and support systems play a vital role in successful implementation. Drawing from experiences in implementing other advanced neuroimaging techniques [14], comprehensive training programs must be developed for radiologists, nuclear medicine physicians, and technical staff. These programs should focus not only on technical operation but also on result interpretation and integration with clinical decision-making.

Cost considerations cannot be ignored in clinical translation. While initial implementation costs may be substantial, including hardware, software, and training expenses, the potential benefits in terms of improved diagnostic accuracy and treatment optimization must be considered. Woo et al. [23] demonstrated that successful implementation of advanced neuroimaging biomarkers requires careful cost–benefit analysis and strategic planning for long-term sustainability.

Quality assurance and regulatory compliance present ongoing challenges that must be addressed. As highlighted by Poldrack et al. [56], maintaining consistent performance and reliability of AI systems in clinical settings requires robust quality management protocols. Regular system audits, performance monitoring, and documentation of outcomes are essential for maintaining regulatory compliance and ensuring optimal clinical benefit.

The success of clinical implementation should be measured through multiple metrics, including diagnostic accuracy, time to diagnosis, and patient outcomes. Vieira et al. [9] emphasized the importance of collecting and analyzing these metrics systematically to demonstrate clinical value and guide continuous improvement. Additionally, staff satisfaction and workflow efficiency should be monitored to ensure successful integration into clinical practice.

Looking forward, the successful translation of these technologies into clinical practice will require close collaboration between researchers, clinicians, and technical experts. As our understanding of Long COVID continues to evolve, flexibility in implementation approaches will be crucial to accommodate new findings and technological advances. The experience gained from implementing AI-driven neuroimaging analysis in Long COVID could provide valuable insights for the broader field of clinical AI applications in neurology and beyond.

## 11. Conclusions

The convergence of artificial intelligence and multimodal neuroimaging offers a promising path forward in understanding and treating Long COVID fatigue. This perspective paper has explored the untapped potential of advanced machine learning techniques, particularly deep neural networks, for dimension reduction techniques in neuroimaging, highlighting how the integration of PET and MRI data analyzed through advanced AI algorithms might reveal previously hidden patterns and associations.

The development of AI-based biomarkers for Long COVID fatigue stands out as a particularly exciting prospect, potentially revolutionizing diagnosis, monitoring, and treatment of the condition. These sophisticated signatures of brain activity and structure might pave the way for more personalized and effective interventions, offering hope for improved outcomes and quality of life for those affected by Long COVID.

However, this path is not without challenges. The technical complexities of integrating diverse data types, the need for interpretable AI models, and the ethical considerations surrounding AI in healthcare all require careful attention. Rigorous validation and replication of findings will be crucial as we push the boundaries of what is possible with AI.

Looking beyond Long COVID, the methodologies and insights developed in this field might help advance our approach to a wide range of neurological and psychiatric conditions. The integration of AI-driven multimodal analysis into Long COVID research represents not just a powerful tool for understanding this specific condition but a paradigm shift in how we study and treat complex brain disorders.

As we move forward, it is essential to keep patient-centered outcomes at the forefront of our efforts. The ultimate goal of these technological advancements is to improve patients’ daily functioning, well-being, and overall quality of life. By embracing this approach while remaining mindful of its challenges and ethical implications, we open the door to a future where our understanding of the brain’s intricacies is matched by the sophistication of our analytical techniques, potentially transforming the lives of those affected by Long COVID fatigue and beyond.
